# In Vitro Experimental Study of Biofiligree^®^ Osteosynthesis in Calcaneus Fracture Fixation

**DOI:** 10.3390/bioengineering13040460

**Published:** 2026-04-14

**Authors:** António Ramos, Olga Noronha, Orlando Simões, José Noronha, José Simões

**Affiliations:** 1 Departamento de Engenharia Mecânica, Universidade de Aveiro, 3810-193 Aveiro, Portugal; a.ramos@ua.pt; 2 ESAD.IDEA—Investigação em Design e Arte, 4450-136 Matosinhos, Portugal; olganoronha@esad.pt (O.N.); orlandojabsimoes@gmail.com (O.S.); 3 Faculdade de Medicina, Universidade do Minho, 4710-057 Braga, Portugal; 4 Venerável Ordem Terceira de São Francisco do Porto, 4050-209 Porto, Portugal; dr.jcnoronha@gmail.com

**Keywords:** Biofiligree^®^, bone fracture healing, osteosynthesis plates, calcaneus prosthesis, patient-centred design

## Abstract

Surgical fixation techniques for bone fracture healing are well established and effective; however, opportunities remain to improve both functional outcomes and the patient experience. The Biofiligree^®^ concept integrates medicine, engineering, and design by reimagining conventional osteosynthesis plates as both therapeutic and aesthetic devices. Inspired by traditional Portuguese filigree, these plates allow patient participation through personalized geometries, patterns, or engravings and may later be transformed into wearable jewellery after removal, preserving them as symbolic artefacts of recovery. This study introduces and biomechanically evaluates a novel calcaneal fixation plate incorporating the biofiligree geometry concept. A biofiligree plate was designed for calcaneus fracture fixation and manufactured in stainless steel 306L. Experimental testing was conducted on synthetic composite calcaneus bone models to simulate anatomical conditions and compare the new design with a standard commercial plate. The biofiligree plate, 2 mm thick, was fixed using five screws and two percutaneous screws positioned at 45° to compress the fracture line. Results demonstrated comparable biomechanical performance between both systems, with similar strain distributions and fracture stabilization. The biofiligree plate showed stresses around 430 MPa and fracture displacement below 0.7 mm. Fixation stiffness values were 1445 N/mm for intact calcaneus, 1065 N/mm for the commercial plate, and 725 N/mm for the biofiligree plate, indicating adequate support for bone healing.

## 1. Introduction

Calcaneal fractures, particularly intra-articular types, represent some of the most challenging injuries in orthopaedic trauma due to the calcaneus’s unique anatomy, its high load-bearing demands, and its articulation with multiple bones [1]. The calcaneus, or heel bone, is the largest tarsal bone and forms the foundation of the hindfoot. It articulates superiorly with the talus at the subtalar joint and anteriorly with the cuboid bone. These articulations enable inversion and eversion of the foot while providing stability during stance and propulsion phases of gait [2]. Surgical expertise is a decisive factor in the outcomes of calcaneal fracture fixation with locking compression plates [3]. Open reduction with plating and closed reduction techniques can yield different results and concluded that plate fixation may be more effective than screw fixation for calcaneal fractures. Intra-articular fractures, which involve the posterior facet of the subtalar joint, account for approximately 75% of all calcaneal fractures and are often caused by high-energy axial loading mechanisms, such as falls from height or motor vehicle collisions. These injuries frequently produce complex comminution and displacement, disrupting the subtalar joint’s congruity and leading to long-term functional deficits if not managed appropriately [4] and reported that early weight-bearing after minimally invasive fixation may cause high stress concentrations and induce calcaneal stress fractures [5].

Prosthetic replacement of the calcaneus, though rare, becomes necessary in cases of severe comminution, chronic osteomyelitis, avascular necrosis, or tumour resection [6,7]. In such cases, conventional fixation methods may fail to provide sufficient stability or restore function, requiring a custom-designed calcaneal prosthesis to reconstruct the hindfoot and restore load-bearing capacity. The calcaneus’s pivotal role in force transmission and complex joint articulation demands implants engineered to closely replicate its anatomy and biomechanical properties. Misalignment or inappropriate material selection can result in altered load distribution, stress shielding, or degeneration of adjacent joints.

Recent advances in 3D printing and biomaterials have enabled the production of patient-specific implants from biocompatible materials such as titanium alloys and composite polymers. These custom prostheses offer high anatomical accuracy, mechanical strength, and porous architectures to promote osseointegration. Early clinical reports suggest promising functional outcomes and implant longevity, but long-term performance, particularly regarding joint preservation, infection resistance, and gait restoration, remains under investigation. Biomechanically, the calcaneus plays a critical role in transmitting body weight from the talus to the ground while acting as a lever arm for the Achilles tendon. Fractures can disrupt this force distribution, producing abnormal load patterns across the foot and ankle complex. Malunion, joint incongruity, or loss of calcaneal height and width can result in altered gait mechanics, chronic heel pain, reduced hindfoot mobility, and, ultimately, post-traumatic subtalar arthritis [8,9]. These complications significantly impair mobility and quality of life.

Advanced tools such as finite element analysis (FEA) and experimental in vitro studies combined with cadaveric and in vitro studies, enable researchers to simulate complex loading conditions and analyse internal stress and strain distributions in the calcaneus and adjacent joints during weight-bearing activities [10,11]. These models are particularly useful for examining the biomechanical effects of intra-articular disruptions, malalignment, and fixation instability, which are often difficult to assess in vivo. Qiang et al. [12] found that the fixation status of the sustentaculum plate screw had minimal effect on the biomechanical performance of the calcaneus. Wang et al. [13] performed a meta-analysis comparing cannulated screw fixation and plate fixation for displaced intra-articular calcaneal fractures, concluding that both yielded similar postoperative functional outcomes.

A key issue in calcaneal biomechanics is the role of percutaneous screws in achieving stable fixation. Percutaneous screw fixation is a minimally invasive technique indicated for selected displaced intra-articular calcaneal fractures, particularly simpler configurations such as Sanders type II and III. Compared with open reduction and internal fixation, this approach offers several advantages, including reduced soft-tissue disruption, lower rates of wound complications, shorter hospital stay, and faster recovery [14,15]. From a biomechanical perspective, properly positioned cannulated screws can restore critical calcaneal parameters such as height, width, and Böhler’s angle, while providing adequate stability for early functional loading in appropriately reduced fractures [16]. Clinical outcomes are encouraging since Meena et al. [17] reported fracture union in all cases, with a mean American Orthopaedic Foot and Ankle Society score of 80 and minimal alignment complications in patients treated with closed reduction and percutaneous screw fixation. More recently, Jiang et al. [18] demonstrated that percutaneous screw fixation yielded radiographic and functional results comparable to open techniques, with the added benefit of fewer soft-tissue complications.

Through FEA or experimental analysis, it is possible to assess how variations in screw placement, plate design, and reduction techniques affect stress concentration and load transfer across the subtalar joint. This knowledge supports surgical planning by promoting fixation constructs that more closely replicate the load-bearing capacity and anatomical behaviour of the intact hindfoot [19] FEA has been used in various orthopaedic studies, Pînzaru et al. [20] evaluated the biomechanical performance of the C-Nail^®^ system compared to conventional plate fixation for displaced intra-articular calcaneal fractures, concluding that the C-Nail^®^ system provides sufficient stability.

Experimental studies have also been extensively used to investigate calcaneal fracture fixation, employing techniques such as strain gauges and digital image correlation. Irwamsyah et al. [21] measured strain changes in the calcaneus stabilized with a 316L plate for Sanders type II fractures, finding good agreement between the two measurement methods. For Sanders type III and IV fractures [22], reported similar clinical results with locked plate osteosynthesis and a T-shaped locking plate for fractures of the anterior process of the calcaneus involving the calcaneocuboid joint [23].

This research raises important questions about the future of implantable devices, bridging cultural heritage, biomechanical engineering, and sustainability by transforming orthopaedic implants into commemorative objects. The intersection of design, mechanics, and cultural identity expands the possibilities for what medical devices can signify, and what they can become, once their therapeutic purpose has been fulfilled. In this context, we evaluated the biomechanical performance of a novel calcaneal fracture fixation prosthesis through experimental modelling, supported by comparative mechanical analysis. Given the calcaneus’s complex anatomy and high mechanical demands, optimizing fixation strategies is essential to restore function and prevent complications such as subtalar degeneration or altered gait.

## 2. Materials and Methods

### 2.1. The Biofiligree Plate Concept

**Figure 1:**
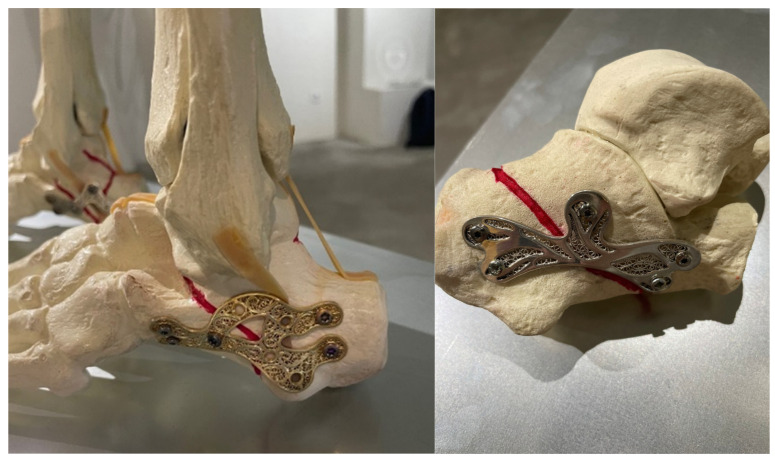
Examples of biofiligree calcaneus bone fixation plates.

A novel concept explored in this study involves the design and development of biofiligree bone fixation plates, merging biomedical engineering with traditional craftsmanship [24]. This approach integrates filigree, a delicate, culturally significant metalworking technique from Northern Portugal, into osteosynthesis design. These “medical jewellery” plates incorporate intricate geometric patterns such as spirals, scales, rhodillions, snails, and cornucopias, fabricated from biocompatible materials including stainless steel and silver (Figure 1). Mechanical testing is essential to evaluate the structural feasibility of these designs for orthopaedic fixation. Identifying configurations that optimize load transfer and minimize stress concentrations within the hindfoot is critical [5,10].

Once bone healing is complete, these plates may be removed and transformed into wearable jewellery such as bracelets, pendants, or rings, symbolising recovery and personal resilience [24]. However, implant removal is not routinely performed after fracture healing and is typically considered only in specific clinical situations, such as patient discomfort, implant-related complications, or patient preference; the proposed design anticipates this possibility without assuming it as standard clinical practice. It is also important to emphasise that Biofiligree plates are primarily intended for low load-bearing fracture applications, where reduced mechanical demands make such designs more suitable.

This paradigm introduces aesthetic, emotional, and symbolic dimensions into medical devices, reframing orthopaedic implants as objects with both therapeutic and post-therapeutic significance. The concept is also applicable to interphalangeal arthrodesis, where the lightweight and fine structure of filigree plates offers potential advantages for load-bearing joints of the foot and ankle. Proof-of-concept work in ulna fracture fixation has demonstrated that filigree plates, when fabricated from surgical-grade materials and designed within appropriate biomechanical constraints, can achieve performance comparable to conventional hardware [24].

### 2.2. The Plate Models for Calcaneus Fracture

**Figure 2:**
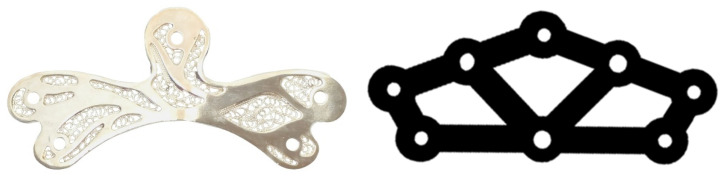

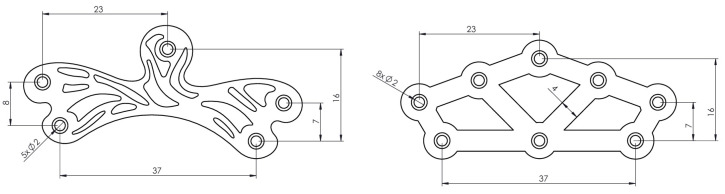
Biofiligree plate and commercial calcaneus bone fracture fixation plates.

For experimental evaluation, calcaneus bone plate specimens (Figure 2) were designed and manufactured. A commercial plate (www.arthrex.com) of equivalent thickness and size was used for comparison, obtained by reverse engineering. Both models had a thickness of 2 mm and employed the same fixation parameters: five cortical screws (2.0 mm diameter, 20 mm length) to ensure consistency and comparison between models with same number of screws and size.

### 2.3. The In Vitro Experimental Calcaneus Model

**Figure 3:**
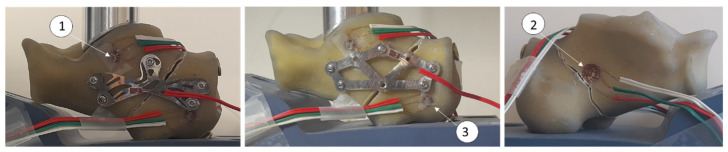
Experimental filigree and commercial plates with strain gauges: (**left**) filigree calcaneus plate; (**middle**) commercial calcaneus plate: and (**right**) intact synthetic calcaneus. 1—rosette placed in the anterior view of the calcaneus with biofiligree plate; 2—strain gauge placed on the posterior view of the calcaneus; 3—rosette placed in the anterior view of the calcaneus wit commercial plate.

A composite Sawbones^®^ calcaneus model (Absolute™ 4th Gen., 17 PCF Solid Foam Core, Large, Washington, DC, USA) was used to perform the experiments, with a surgically created midsection fracture stabilized by an experienced surgeon to reproduce a clinical scenario. The plates were fabricated from 316L stainless steel and fixed with five titanium screws plus two percutaneous screws and presented in Figure 3 with the experimental assembly. A custom polyurethane base produced by CAM (computed add manufacturing) technology supported the calcaneus at 20° from the horizontal, ensuring reproducible positioning with a cavity to put in place the calcaneus bone. A loading rod (20 mm diameter) was contoured to match the calcaneus surface, enabling uniform load distribution as the same surface of the calcaneus contact.

Strain was measured using contact strain gauges (model KFG-1-120-D17-11 L3M2S, Kyowa Electronic Instruments, Tokyo, Japan). Three rosettes were placed, two in the lateral face on cortical bone near the plate, the other in the medial face near the fracture line and one gauge was placed on the plate itself (Figure 3) to evaluate the load in plate. Loading was applied using a Shimadzu 10 kN machine (Kyoto, Japan), with vertical loads of 200, 300, and 350 N applied at 5 mm/min. Each step was held for 10 s to ensure stable and repeatable measurements.

## 3. Results

### 3.1. Comparison of Experimental Strains in Rosettes

**Figure 4:**
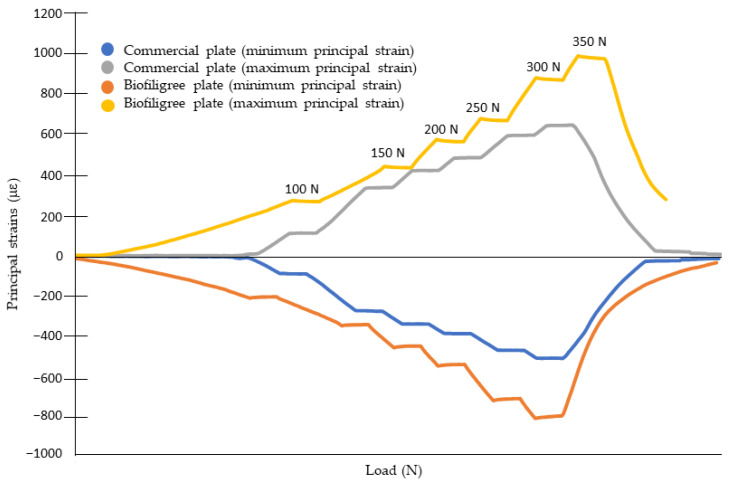
Maximum and minimum principal strains at rosette 3 up to 350 N for the biofiligree and commercial plates.

Table 1 summarizes the key experimental results. Strain measurements demonstrated high repeatability, with deviations remaining below 5% across five trials. Figure 4 presents an example of the maximum and minimum principal strains evolution at rosette 3 under loads up to 350 N for both the biofiligree and the commercial plate. Strain gauge data indicated higher cortical strains with the biofiligree plate compared to the commercial plate under identical loading conditions. At rosettes 1 and 3 (Figure 3), cortical surface strains were the lowest across all models. For rosette 3, positioned beneath the plate, the differences between the biofiligree and commercial plate were 2% for maximal principal strains and −17% for minimal principal strains; for rosette 1, the respective differences were −15% and 3%.

Rosette 2, located on the opposite face of the calcaneus, recorded the highest strain values, primarily in compression, and was identified as the critical region after fracture. Both plates increased cortical strains around the rosettes; however, the biofiligree plate produced the most pronounced effect, with cortical strains rising by 126% in compression and 138% in tension compared to the intact calcaneus. The differences in maximal and minimal strains between the two plates were −120% and −405%, respectively.

### 3.2. Experimental Fixation Stiffness

**Figure 5:**
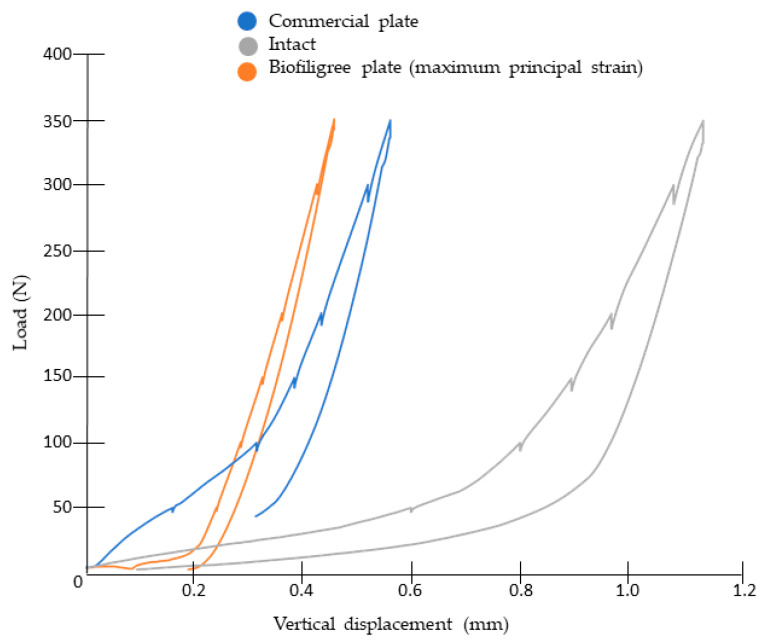
Experimental measurements of fixation stiffness (load vs. displacement).

Fixation stiffness is an important biomechanical indicator, measured as the relationship between applied force and displacement. Experimental fixation stiffness was determined from the load-displacement response recorded during mechanical testing and calculated as the slope of the linear (elastic) region of the curve (N/mm). Figure 5 illustrates the model’s behaviour during loading and unloading. Compared to the intact calcaneus, both plates exhibited reduced stiffness. The response included an initial accommodation phase—longer for the filigree plate—followed by recovery upon unloading. The stiffness of the intact calcaneus was 1445 N/mm, while the commercial and filigree plates demonstrated values of 1065 N/mm and 725 N/mm, respectively. Notably, the stiffness of the commercial plate was closer to that of the intact calcaneus, with a difference of 26%, whereas the filigree plate showed a difference of approximately 50%.

### 3.3. Experimental Influence of Percutaneous Screws

**Figure 6:**
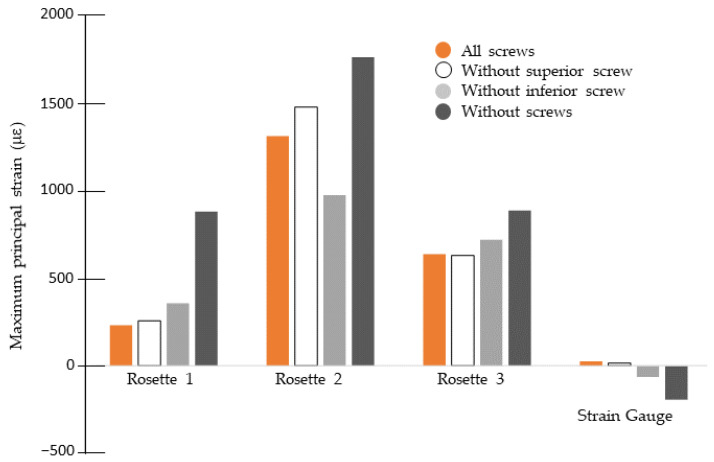
Influence of percutaneous screws on strain distribution with the biofiligree plate.

Percutaneous screws influenced the strain distribution measured in both the calcaneus (via rosettes) and the plate (via strain gauges). Figure 6 shows the principal bone strains recorded by the rosettes alongside the strains measured on the surface of the filigree plate. The superior percutaneous screw had minimal effect on the strains in the fractured calcaneus with the filigree plate. A slight increase was observed at rosette 2, which, as noted previously, exhibited the highest strain values. Removal of both percutaneous screws resulted in higher strains across all three rosettes. These results indicate that the use of the inferior percutaneous screw provides a biomechanical benefit. The use of percutaneous screws changes the strains in the plate, for compression instead of traction with all screws.

## 4. Discussion

The integration of biofiligree patterns into osteosynthesis plates represents a novel intersection of biomedical engineering, cultural heritage, and patient-centred design. This study evaluated the mechanical performance of a filigree-patterned calcaneal fixation plate compared to a conventional commercial plate. Both devices withstood the applied loads without material failure. Experimental strain measurements revealed that the filigree plate exhibited slightly higher strain values, and its relative displacement at the fracture line was approximately 5% greater than that of the commercial plate. Despite this marginal difference, both plates provided comparable structural stability, suggesting that the filigree design does not compromise the mechanical integrity required for effective fracture fixation.

These outcomes align with previous studies showing that decorative or patterned designs can be incorporated into bone fixation devices without significantly reducing mechanical performance [24]. Many available studies are retrospective case series or nonrandomized comparisons; while recent systematic reviews support the safety and efficacy of percutaneous screw fixation in selected displaced intra-articular calcaneal fractures, higher-quality randomized trials and long-term follow-up are still limited [25]. Surgeons should interpret comparative advantages (e.g., lower wound complications) in the context of local expertise, available imaging, and patient comorbidities.

The present study provides new insights into the distribution of cortical strains in the calcaneus following fracture fixation with commercial and biofiligree plates. The results also echo findings from recent work on screw configuration: for example, a biomechanical comparison of different screw placements within calcaneal fixation systems showed that screw orientation, number, and positioning significantly affect primary stiffness and load transfer [26]. Stress concentrations consistently occurred around screw holes, reinforcing the critical influence of screw–plate mechanics on implant behaviour.

Strain gauge and rosette measurements confirmed that fixation alters the physiological load transfer of the calcaneus, with the most pronounced changes observed at rosette 2, located on the opposite face of the fracture (medial face). This region consistently recorded the highest strain values, primarily in compression, and can therefore be identified as a biomechanical hotspot after fixation. Similar findings have been reported in computational models, where posterior and plantar aspects of the calcaneus were shown to experience elevated stress and strain following intra-articular fractures [27,28]. These localized strain concentrations are clinically significant, as they may predispose the bone to microdamage, delayed union, or secondary complications.

Comparisons with other biomechanical investigations of calcaneal fixation devices further support our findings. Richter et al. [29] showed that locking plates reduce micromotion compared to non-locking designs; Sato et al. [30] found that locking plates better maintain anatomical alignment; and Yu et al. [27] demonstrated that anatomical plate designs can improve stress distribution and fixation stability. In our model, fracture displacement under load (approx. 0.65 mm) was between values reported in these earlier studies, underscoring that the biofiligree plate approximates conventional solutions.

Concerning the analysis of results between the filigree and commercial plates, it highlighted the role of implant design in modulating strain distributions. Both plates caused increased cortical strains compared to the intact calcaneus, but the biofiligree plate amplified strain magnitudes to a greater extent. This outcome likely reflects its modified geometry, which shift more load to the underlying bone. Such findings are consistent with finite element analyses showing that plate stiffness and contour directly influence strain levels and stability in the calcaneus [27,31]. While elevated strains can, in some cases, promote bone healing through mechanotransduction, the increases observed with the biofiligree plate, although reaching up to 138% above the intact condition in tension, remain relatively low in absolute magnitude and therefore are unlikely to pose significant risks of implant loosening or cortical resorption.

Rosette-specific results further revealed heterogeneity in strain responses. At rosettes 1 and 3, positioned closer to the fixation site, strain differences between the two plates were modest, whereas at rosette 2 the disparities were more pronounced. This site-specific behaviour reinforces the importance of evaluating fixation performance not only in terms of global stiffness but also in localized strain patterns. Previous studies have similarly emphasized that fixation constructs redistribute stresses unevenly across the calcaneus, with local stress concentrations being highly sensitive to plate geometry and screw configuration [12,31].

A key clinical advantage of percutaneous screws is preservation of soft-tissue envelope and periosteal blood supply. This is particularly important in calcaneal fractures because the lateral soft tissue is vulnerable to wound breakdown. Lower rates of infection, wound dehiscence, and need for reoperation have been repeatedly reported for minimally invasive screw techniques compared with extensile lateral approaches. For patients with soft-tissue compromise (smoking, diabetes, peripheral vascular disease) or when early surgery is necessary, percutaneous fixation can reduce surgical morbidity [32].

Percutaneous screw fixation has become an established minimally invasive option for selected displaced intra-articular calcaneal fractures, particularly Sanders type II. The approach aims to restore articular congruity and calcaneal geometry while minimizing soft-tissue dissection and wound complications associated with extended lateral approaches. Several clinical series and comparative studies report that, for appropriately selected Sanders II fractures, percutaneous screw fixation provides functional and radiographic outcomes comparable to open reduction and internal fixation, with lower rates of wound healing problems [33].

Biomechanically, well-placed percutaneous screws can re-establish stability of the posterior facet and lateral wall and help maintain Böhler’s angle and calcaneal height under physiologic loads [34]. Finite element and biomechanical studies suggest that percutaneous constructs can achieve sufficient stiffness for early functional rehabilitation in Sanders II fractures, although absolute mechanical stability may be lower than with plate fixation in some configurations. The practical implication is that screw constructs can be adequate when the fracture pattern allows for anatomical reduction and compression across key fragments [34].

However, the technique has limitations and is technique-sensitive. Percutaneous fixation generally requires: a fracture morphology amenable to closed or minimally open reduction (i.e., a single or simple split of the posterior facet typical of Sanders II); reliable imaging (fluoroscopy or intraoperative CT) to confirm reduction; and appropriate screw trajectories to control the tuberosity, sustentacular fragment and posterior facet. Complex multi-fragmentary patterns (Sanders III/IV type) or large lateral wall impaction may require open reduction and internal fixation with a plate for direct visualization and buttressing. Several comparative studies caution that when an inadequate reduction is accepted for the sake of minimal invasiveness, long-term subtalar arthritis or malalignment may worsen functional outcomes [14].

Clinical outcomes and decision making therefore depend on careful patient and fracture selection. Percutaneous screw fixation is most suitable when closed/minimally invasive techniques can reliably restore the posterior facet and overall calcaneal height/width and when the surgeon can achieve stable interfragmentary compression. For Sanders II fractures meeting these criteria, percutaneous techniques can shorten operative time, reduce hospitalization and lower wound complication rates while delivering comparable midterm functional scores. Ongoing randomized and large comparative trials are refining the relative indications and long-term outcomes [18].

Beyond mechanical performance, the biofiligree plate introduces patient-centred and symbolic dimensions. By allowing patients to choose decorative patterns, then transforming the removed plate into jewellery post-healing, the design fosters emotional engagement, resilience, and a sense of ownership in the recovery process. Studies in patient-centred design show that personalization, aesthetics, and symbolic meaning in medical devices or environments can positively influence patient satisfaction, perceived well-being, and adherence to treatment [35,36]. This cultural and emotional integration amplifies the device’s value beyond purely biomechanical outcomes.

Together, our findings suggest that fixation stability can be maintained across diverse plate architectures, whether conventional, anatomical, biofiligree, or even aesthetic, so long as key mechanical elements like screw configuration are preserved. The biofiligree approach demonstrates that orthopaedic implants can simultaneously meet functional, mechanical, and emotional needs, marking a meaningful step toward more holistic, patient-centred fracture care.

Some limitations must be acknowledged. This study did not include long-term or fatigue testing, as the experimental protocol focused on initial mechanical behaviour under controlled conditions and did not account for cyclic loading, material fatigue, or long-term structural stability. In addition, locally reduced thickness regions in the Biofiligree plate may introduce stress concentrations that could act as potential crack initiation sites, increasing susceptibility to fatigue-related failure. While synthetic bone models are suitable for controlled mechanical testing, they do not replicate the full complexity of human bone, including heterogeneity, anisotropy, and biological responses. Consequently, further research should incorporate fatigue analysis, fracture assessment, and long-term biomechanical evaluation, as well as possible in vivo studies to assess corrosion behaviour, osseointegration, and overall clinical safety. Future work should also combine experimental strain measurements with finite element modelling to refine predictions of local mechanical behaviour and include clinical follow-up studies to determine whether the observed strain patterns influence healing outcomes or complication rates. Additionally, user-related aspects, such as patient preferences, ease of adoption, and the potential impact of decorative aesthetics on treatment acceptance, should be explored, alongside optimisation of decorative geometries to balance mechanical performance with aesthetic and emotional value.

## 5. Conclusions

The transformation of osteosynthesis plates into bio-jewellery, wearable artefacts preserved after fracture healing, introduces a novel dimension to orthopaedic care. This paradigm shifts the role of fixation devices beyond mechanical stabilization to encompass patients’ psychological and emotional well-being.

By allowing individuals to retain a tangible and meaningful symbol of recovery, the biofiligree plate fosters resilience, identity, and a personal connection to the healing process. The biofiligree plate evaluated in this study not only demonstrated biomechanical performance comparable to conventional fixation systems but also embodied an innovative, patient-centred design philosophy.

Through the integration of biomedical functionality with cultural heritage and aesthetic expression, this approach enriches the patient experience and offers a redefinition of how medical devices can contribute to both physical and emotional recovery.

## Figures and Tables

**Table 1:** Comparison of experimental strains in bone and plate.

	Rosette 1		Rosette 2		Rosette 3		Gauge
	Maximal Principal Strain (με)	Minimal Principal Strain (με)	Maximal Principal Strain (με)	Minimal Principal Strain (με)	Maximal Principal Strain (με)	Maximal Principal Strain (με)	(με)
Intact calcaneus	206.3 ± 1	−156.6 ± 2	550.3 ± 3	−980.3 ± 2	285.2 ± 4	−202.0 ± 2	-
Filigree plate	230.9 ± 2	−213.9 ± 2	1310.0 ± 5	−2211.0 ± 1	636.3 ± 4	−420.7 ± 2	−27.1 ± 5
Difference to intact (%)	12%	37%	138%	126%	123%	108%	-
Commercial plate	201.5 ± 4	−208.6 ± 5	595.4 ± 9	−438.1 ± 2	649.3 ± 1	−509.4 ± 6	−160.0 ± 5
Difference to intact (%)	−2%	33%	8%	−55%	128%	152%	-
Difference between plates (%)	15%	−3%	−120%	−405%	2%	−17%	-

## Data Availability

The data presented in this study are available on request from the corresponding author.
